# Weldon, Bateson, and the origins of genetics: Reflections on the unraveling and rebuilding of a scientific community

**DOI:** 10.1371/journal.pgen.1010379

**Published:** 2022-10-27

**Authors:** Lea K. Davis

**Affiliations:** 1 Division of Genetic Medicine, Department of Medicine, Vanderbilt University Medical Center, Nashville, Tennessee, United States of America; 2 Vanderbilt Genetics Institute, Vanderbilt University Medical Center, Nashville, Tennessee, United States of America; 3 Department of Psychiatry and Behavioral Sciences, Vanderbilt University Medical Center, Nashville, Tennessee, United States of America; 4 Department of Biomedical Informatics, Vanderbilt University Medical Center, Nashville, Tennessee, United States of America; 5 Department of Molecular Physiology and Biophysics, Vanderbilt University, Nashville, Tennessee, United States of America; University of California Los Angeles, UNITED STATES

“*Science*, *like most forms of human activity*, *is occasionally liable to lose sight of its ultimate ends under a flood of controversy*, *the strugglings of personal ambition*, *or the fight for pecuniary rewards or less physical honors …*

*But science, no less than theology or philosophy*, *is the field for personal influence, for the creation of enthusiasm*, *and, for the establishment of ideals of self-discipline and self-development.”—Karl Pearson [1]*

More than a century has passed since the publication of R.A. Fisher’s 1918 paper “The correlation between relatives on the supposition of mendelian inheritance”. Celebrated across the world as a major turning point in the young science of genetics, the paper formally reconciled mendelian and biometric approaches to inheritance by introducing the concepts of variance and polygenicity and laying the groundwork for the complex trait liability threshold model, which remains highly relevant to modern human genetics [2]. While many in the field of human genetics are aware of this work, few are aware of the controversy that preceded it. It is an origin story for the field of human genetics, filled with both drama and discovery. The lessons herein resonate today as we continue to contend with the trials of hyper-competitive incentive structures, a workaholic academic culture, and larger-than-life egos that often hide the vulnerability we feel as mere mortals tasked with tremendous feats.

Now is an opportune time to revisit these origin stories of our field. Given the magnitude of the problems facing us today, there is an urgent need for larger and larger scientific communities to function healthfully. Anyone who has felt in their gut the awe and humility of discovery understands that we are not *entitled* to learn the secrets of the Universe, we *labor* to gain even the smallest insight. Individually, we will be wrong far more often than we will be right. In this tale, what began as a scientific debate between friends devolved into a bitter argument, creating a chasm in the scientific community that persisted for decades and arguably stalled the progress that Fisher’s paper later spurred. In a sense, this story can be viewed as a “morality tale” about the dangers of competitiveness, the responsibility of influential voices, and the importance of maintaining a profound sense of humility as a scientist. The following centers the very elements that complicate healthy team science—friendship, ego, and ambition, and illustrates how they were weaponized in a war between scientists that was said to have resulted in at least 1 casualty [3–6]. It is my hope that by shining a spotlight on the interpersonal aspects of doing science, we may deepen our appreciation for the profound and enduring impact that our interactions can have on the scientific questions we choose to answer, the methods we adopt, and how we conceive of “ground truth.”

The story began long before Fisher was born, in 1859, with the publication of Charles Darwin’s book On the Origin of Species [7]. Darwin, privileged by wealth and education, traveled widely at an early age and compiled detailed notes on flora and fauna across the world. He gained an international reputation when he introduced the scientific world and the lay public to his theories of evolution and natural selection. Darwin’s half-cousin, Francis Galton, who was interested in similar ideas, had also recently published a book on his travels titled “Narrative of an explorer in tropical South Africa.” Despite a Royal Geographic Society review claiming the book was written with much “manly humor and style” [8], it was no comparison to Darwin’s thesis. After the publication of his cousin’s book, Galton (who later became known as the “father of eugenics”), focused his attention on questions pertaining to human variation, heredity, and selection provoked by Darwin’s theories of evolution. During his career, he formalized the statistical concepts of correlation and regression and his influence loomed large on all the personalities involved in the conflict to come.

Twenty years after the publication of Darwin’s book (1879), the 2 most important players in this drama, William Bateson (1861 to 1926) and Walter Frank Raphael Weldon (1860 to 1906), met as undergraduates at the University of Cambridge. Despite their different backgrounds, the two shared a common interest in zoology and both made his home in academia. Bateson, whose father was Master of St. John’s College (a constituent college of the University of Cambridge), was on track to attend Cambridge since childhood. In contrast, Weldon was the first in his family to attend a University, though his father Walter Weldon was a celebrated chemist and innovator [9]. Weldon quickly developed a reputation in the zoology department for being a clear and insightful teacher. During their years at Cambridge, a friendship grew, and though he was only 1 year older, Weldon became a peer mentor to Bateson.

By this time, the theory of evolution was widely accepted within scientific circles; however, the mechanisms through which new species arose remained a great mystery and was one of the most compelling scientific questions of the day. Bateson and Weldon were captivated and each set out to understand the process of speciation. Both men were also highly influenced by Galton’s work which, by then, had provided much of the foundation for modern statistics. Further, they were both personally acquainted with Galton and the three corresponded regularly. During the next decade, Bateson, Weldon, and Galton theorized widely on whether speciation was due to a slow and continuous process of selective breeding or could occur due to a major and rapid biological change (i.e., saltation).

Weldon and his wife Florence (nee Tebb) began collecting and studying wild populations of shrimps and crabs hoping to understand the relationship between continually varying morphological features within and between species. Weldon was quantitatively minded but lacked in computational skill [10]. Florence, who graduated from Girton College, Cambridge, was schooled in computation and a rigorous analyst. Together they collected, measured, and recorded dozens of anthropometric features on hundreds of samples, and Florence computed hundreds of descriptive statistics. Eventually, they sought a collaboration with Karl Pearson upon his joining the faculty of the University College London in 1892. It was their data on wild crab populations and questions about the continuous characteristics of these populations that provided Pearson both the means and motivation to develop the statistical methods for which he eventually became famous. Pearson once said, “Any mathematician could have done what I have done, a dozen or so better, especially if they had suggestions from Weldon almost daily at lunch for 4 or 5 years” [11–13]. Through years of painstaking data collection and analysis, the team eventually began to realize that anthropometric traits that distinguished *different* crab species also varied greatly *within* species. Indeed, upon the collection of enough data, even “dimorphic” traits appeared continuous. Thus, they came to believe that the most likely mechanism to explain evolution was slow and gradual natural selection.

Furthermore, Weldon believed the theoretical proof of this biological process could only be achieved through mathematics claiming that “The questions raised by the Darwinian hypothesis are purely statistical, and the statistical method is the only one at present obvious by which that hypothesis can be experimentally checked” [14]. This was not to say that he found no use for experimental data in uncovering potential mechanisms, only that he firmly believed in the necessity of statistical approaches to populations [15]. Nevertheless, this was not a popular opinion. As Pearson later remembered, “The very notion that the Darwinian theory might after all be capable of statistical demonstration seemed to excite all sorts and conditions of men to hostility. Weldon, instead of being allowed to do his own work in his own way, had to be constantly replying to letters, some even 18 sheets long … These letters were not sympathetic and suggestive, but mostly purely controversial,” [1]. Nevertheless, Pearson continued to work with Raphael and Florence Weldon and presented ideas that emerged from the crab populations including the concepts of standard deviation, covariance, and coefficient of variation [16–19]. It was also through Weldon that Pearson eventually met Galton. Galton became a life-long mentor and friend to Pearson and the two became so close that after Galton died, Pearson spent 20 years writing his definitive biography [20].

It should be noted that while Weldon believed in the necessity of statistical evidence to confirm observations, particularly in wild populations, he also valued laboratory experimentation and mechanistic understanding. In addition to his work on wild populations, he collaborated on breeding experiments using Japanese waltzing mice to evaluate segregation patterns in coat color, among other traits. Indeed, unpublished manuscripts (“*The Theory of Inheritance*”) written by Weldon in his later life [21] attempted to bring statistical and mechanistic insights gained through analysis of both wild populations and experimental studies together into a unified theory [15,21].

Meanwhile, Bateson worked diligently to perform carefully controlled experimental crosses of plant species in the Cambridge botanical gardens and frequently observed segregation of traits that clearly behaved as dimorphic (e.g., petal color). Based on these observations, he became convinced of the potential for “sporting mutations” (i.e., rare alleles with large phenotypic impacts) to drive evolutionary leaps. Bateson faced his own share of political pressure aimed at his theories and methods. The Director of the Morphological Laboratory at Cambridge, Adam Sedgwick, did not think highly of Bateson’s work. In a letter to his sister, Bateson confessed that “Sedgwick tells me he would not wish me to have Weldon’s lectureship if W. goes to University College. He says, as I expected, that I have gone too far afield and that my things are ‘a fancy subject’” [22].

The situation at Cambridge worsened for Bateson as he began expanding his research group. Bateson was sympathetic to the women’s suffrage movement in which his mother Anna Aitkin and sisters Margaret, Anna, and Mary Bateson were prominently involved (Box 1). He welcomed women into his laboratory group. Some of the first papers from his group were coauthored by his sister Anna who also worked with Francis Darwin (botanist and son of Charles Darwin) during her studies at Cambridge. However, Sedgwick strongly disapproved of women in the academy, and this principled position cost Bateson throughout his career. Though he was employed at Cambridge for the entirety of his career, his promotion was overlooked for more than a decade. Nevertheless, his integrity remained intact and his collaborations with many female scientists including Edith Saunders, later referred to as the “mother of British plant genetics” [23], were incredibly productive (Box 2).

**Box 1. The price of feminism**

We would be amiss to neglect the larger social context in which Weldon, Bateson, and their female colleagues were working. Women’s suffrage was a major political debate that impacted the career trajectory of men and women who supported women’s right to vote. In 1906, Mary Bateson was one of nearly a dozen women and men comprising a Women’s Suffrage Deputation who petitioned the Prime Minister from the floor of the House of Commons. Mary spoke on behalf of “women who are Doctors of letters, science, and law in the Universities of the United Kingdom and of the British Colonies ….who believe the disenfranchisement of one sex to be injurious to both and a national wrong in a country which pretends to be governed on a representative system”. (Women’s Suffrage Deputation, May 19, 1906; Received by the Prime Minister, Sir Henry Campbell-Bannerman, published by the National Union of Women’s Suffrage Societies, 25 Victoria Street, Westminster London, S.W.)

**Box 2. Note on the important contribution of Edith Rebecca Saunders to the early discipline of genetics**

Edith Rebecca Saunders was an influential early geneticist. Her experiments led to the characterization of the “allelomorph” (i.e., heterozygote and homozygote), many decades before Rosalind Franklin’s groundbreaking work characterizing the structure of DNA. She served as the Vice President of the Linnean Society, the 1920 President of the Botanical Section of the British Association for the Advancement of Science, and the 1938 President of the Genetical Society.

During these years, Weldon and Bateson corresponded often to debate the merits of gradual selection versus sporting mutation as well as the best methods of evaluation. In an 1888 letter, Weldon wrote to Bateson, “I have not written to you for a long time because I have the spirit of polemic upon me: and I have wished to consider carefully the words I should say to you. In the first place, I will tell you 3 sets of things which ought as it seems to me to annoy you …” He ended the letter lightheartedly by saying, “And when are you coming to crush me???” [6]. The friendly teasing in these correspondences foreshadowed the break that was to come.

In 1894, Bateson published the book “Materials for the study of variation treated with especial regard to discontinuity in the origin of species”. He presented the experimental crossing methodology that his team employed and used it to argue the case for the importance of saltation. Weldon was asked to write a critique of the book for *Nature*, and this seemed to mark the beginning of the public end to their friendship. While it was considered an overall positive review, he criticized Bateson’s thesis of evolution by saltation. Weldon was unimpressed by Bateson’s arguments stating “If the criticism and enunciation of opinions had been performed with the same care as the collection of facts, the commentary which runs through the book would have gained in value, and several inaccuracies, partly due to want of acquaintance with the history of the subject, would have been avoided” [24]. Bateson’s next move signaled that he was both hurt and angry. He turned to Galton to vent his frustration in a series of letters that heavily criticized Weldon’s analysis of wild crab populations [25].

A year later, the situation intensified when William Turner Thiselton-Dyer, Director of the Royal Botanic Gardens in Kew, wrote a letter to the editor of *Nature* in which he praised work recently presented by Weldon and Pearson stating, “I entirely agree with him in minimizing the value of ‘sports’ in evolution” [26]. This drew severe criticism from Bateson who responded the next month with his own letter to *Nature* announcing that he “…ventured to deal with this case because it seems to be generally supposed by those not acquainted with the facts, that the origin of the modern florists’ flowers has in general been very gradual”. Eventually, Weldon also weighed in, arguing that Bateson omitted pertinent information in his letter and that his “emphatic statements are simply evidence of want of care in consulting and quoting authorities referred to.” Bateson furiously replied, “Upon what grounds [Weldon’s] statement has been made the reader shall now learn, not perhaps without astonishment”. Weldon finally ended the quarrel saying, “Enough has been said to show that Mr. Bateson’s original evidence does in fact bear the interpretation I put upon it…Having done this, my interest in the matter ends, and I do not propose to speak further upon it” [27]. The playful argumentativeness that characterized their earlier written exchanges transformed into bitter antagonism as each became more entrenched in his own methodology and results and suspicious of the other. Eventually, Weldon wrote privately to Bateson saying, “Dear Bateson, I can do no more. First, you accuse me of attacking your personal character, and when I disclaim this, you charge me with a dishonest defense of someone else…If you insist upon regarding any opposition to your opinions concerning such matters as a personal attack upon yourself, I may regret your attitude but I can do nothing to change it” [28].

For the next few years, Bateson, Saunders, and Punnett continued to selectively breed plants and publish observations of discontinuous traits while the Weldons’ continued to gather large amounts of data from wild populations of crabs and collaborate with Pearson in London to develop statistical methods of analysis. The next plot point in the story occurred at the turn of the century when Mendel’s work was “rediscovered.” Hugo de Vries, a botanist from Austria, who was aware of Mendel’s work, attended the 1899 conference of the Royal Horticultural Society where he heard a lecture by Bateson describing the breeding experiments that he and Saunders were performing on flowering plants in Cambridge. Realizing the similarity to Mendel’s earlier pea plant experiments, De Vries published a paper in 1900 referring to the original work of Mendel in a footnote. The year 1900 marked the “rediscovery” of Mendel’s laws by De Vries, Correns, and Tschemark, and gave Bateson the independent evidence that he was seeking (Box 3). He became a huge supporter of the “mendelian” theory and again, wrote to Galton, saying “In case you may miss it… Mendel’s work seems to me one of the most remarkable investigations yet made on heredity, and it is extraordinary that it should have got forgotten.” [29]. Bateson became an ardent supporter of the mendelian model of inheritance, and in 1909, Bateson translated Mendel’s work from the original German into English. Incidentally, he also named his youngest son Gregory after Gregor Mendel.

**Box 3. Recent interpretations challenge the traditional account of the rediscovery of Mendel’s work**

Shan argues that rediscovery is the wrong term to describe the use of Mendel’s work in the subsequent efforts of de Vries, Correns, and Tschemark. All 3 reported Mendel’s observations, but then went on to extrapolate from his work ideas that supported their own discoveries. For example, Shen argues that De Vries, not Mendel, proposed the ideas of dominance and segregation of alleles that he supported with the ratios reported by Mendel [15].

But Pearson and Weldon were skeptical of mendelian ratios believing the results were “too good to be true.” Weldon noted that in the wild, peas often varied more continuously from yellow to green. He argued that Mendel’s results may have been an artifact of the extensive inbreeding required to obtain the pure green and yellow lines prior to the hybridization experiments. His skepticism of mendelian ratios was fed by a larger concern over what he perceived as a lack of quantitative rigor in the field. In the year before Mendel’s work was rediscovered, he wrote to Pearson saying, “The contention ‘that numbers mean nothing and do not exist in Nature’ is a very serious thing, which will have to be fought. Most other people have got beyond it, but most biologists have not. Do you think it would be too hopelessly expensive to start a journal of some kind?” Weldon and Pearson (in consultation with Galton) subsequently established the journal *Biometrika* that produced its first issue in 1901. By 1902, the two schools of thought were cemented and colloquially named, the Mendelians (Bateson, Saunders, and Punnett) in Cambridge, and the Biometricians (Weldon and Pearson) in London. By 1904, about 15 years after the argument first began, civility was so eroded that the editor of *Nature* refused to publish any more letters between Weldon and Bateson on the issue of saltation versus continuous selection.

Eventually, the tension erupted in person at the 1904 meeting of the zoological section for the British Association for the Advancement of Science. The meeting was held at Cambridge, the Mendelians’ turf, and Bateson was the president of the zoological section of the society. It was no surprise that he used his presidential address to lecture on the controversy saying, “For if any one will stoop to examine Nature in those humble places… he will not wait long before he learns the truth about variation… Again and again the circumstances of their occurrence render it impossible to suppose that these striking differences are the product of continued selection, or, indeed, that they represent the results of a gradual transformation of any kind” [30]. Bateson’s words provoked an argument with Weldon on the spot. The argument grew heated and according to reports of the session, conference goers who at first were shifting uncomfortably in their seats eventually began to congregate around the embattled leaders of each side. Finally, the chair of the session, who could not subdue the animated crowd announced in exasperation “Let them fight it out!” [31]. The event was so explosive that 2 years later, when Weldon died suddenly of pneumonia, the New York Times described the scene in his obituary saying that “The debate, which was conducted before a large and somewhat agitated audience, resolved itself into a dialectical dual between the president of the section [Bateson] and Professor Weldon, and developed quite a considerable amount of heat” [32].

Over the next 2 years, the argument overtook both men. Weldon was seemingly obsessed with disproving what he perceived as a narrow interpretation of the mendelian hypothesis of inheritance (particularly dominance) in each subsequent paper in which it was proposed. His last work, “*Theory of Inheritance*,” an unpublished book with 6 manuscript chapters (historical documents held at University College London), incorporated experimental data testing mechanistic hypotheses underlying his statistical observations [21]. Shan [15] and others [33] argue that this manuscript presents a unified theory which may indeed have been the first attempt to reconcile Biometry and Mendelianism. He worked excessively long hours day after day and for many months refused a break, saying “I really want a holiday, but I cannot leave this thing unsettled” [4]. Pearson was concerned and eventually persuaded Weldon to take a vacation. The families adjourned to the English seaside for the Easter break, and it was there that Weldon caught a chest infection that developed into pneumonia. He died a few weeks later at the young age of 46. Pearson, and many others who knew Weldon, believed his premature death was in part due to frenzied overwork and stress that left him physically weakened and unable to fend off the infection. Of his friend, Pearson said: “He was by nature a poet, and these give the best to science, for they give ideas” [1].

After Weldon’s death, the raw bitterness of the Mendelian–Biometrician debate gave way to a quieter resentment that seethed between Pearson and Bateson for another decade, and Weldon’s manuscript book remained in archives. Bateson found that his influence was profound in the United States where mendelian genetics took firm root while London remained the stronghold of the Biometricians. Mendelian genetics caught on early and spread rapidly in the US, in part due to promotion by eugenicists who used genetic determinism to mobilize a racist and classist agenda that permeated US genetics and governmental policy until well after World War II. Later, after distancing genetics from eugenics, theories of mendelian inheritance continued to be the primary model taught in the US. Indeed, the consequences of this community divide can still be seen today in a comparison between UK and US genetics school curriculum.

Twenty-six-year-old Ronald Aylmer Fisher entered the fractured field in 1916, a decade after Weldon’s passing. By this time, the division between the Biometricians and the Mendelians was older than Fisher himself and had profoundly shaped the new field of genetics and an entire generation of zoologists. Fisher was in an ideal position to bridge the divide. Trained as a mathematician but working at Cambridge, the home of the mendelian experimentalists, Fisher was open to the possibility of a simple third solution that unified the observations of both sides. His paper describing this solution was first communicated by Leonard Darwin (Charles’ nephew) to the Royal Society in 1916. The paper included extensive mathematical proofs supporting the central thesis that “In general, the hypothesis of *cumulative* mendelian factors seems to fit the facts very accurately.”

The paper represented the potential for a formal reconciliation between the positions of the Biometricians and the Mendelians. Reginald C. Punnett and Pearson, who had been so close to the heart of the conflict between Bateson and Weldon, were asked to review. The paper was heavily mathematical and from Punnett’s perspective, too theoretical. Of the thesis, Punnett stated “I do not feel that this kind of work affects us biologists much at present. It is too much of the order of problem that deals with weightless elephants upon frictionless surfaces, where at the same time we are largely ignorant of the other properties of the said elephants and surfaces” [34]. Pearson’s review, which he started by acknowledging he was “overfussed with other work,” was similarly dismissive and asserted that the paper was of little interest to the mathematically minded and unlikely to be persuasive to biologists. He closed by stating that “Whether the paper be published or not should depend on mendelian opinion as to the correspondence of the authors hypotheses with observation, and the probability that mendelians will accept in the near future a multiplicity of independent units not exhibiting dominance or coupling” [34]. Though it was not formally rejected, the paper was considered too low impact by the journal and was eventually withdrawn [34]. It was published 2 years later, in 1918, in the Transactions of the Royal Society of Edinburgh. Today, “The correlation between relatives on the supposition of mendelian inheritance” is considered one of the most influential papers in human genetics as it provided a foundation from which the polygenic model of disease and many sophisticated molecular and quantitative approaches subsequently emerged.

However, it would still take many years of healing before the human genetics community truly integrated these ideas into praxis. As the field of medical genetics emerged, work in the US was almost exclusively focused on single-gene disorders for decades. The biometrician view of polygenicity and continuous distributions of genetic liability, which became a staple of genetics curriculum in Europe, was less frequently taught in US classrooms. Even today, many genetics students reach graduate school in the US without ever having been exposed to Fisher, Weldon, or a polygenic model of disease. In contrast, even most US middle school students have heard of Mendel’s principals of inheritance. The comparative lack of quantitative genetics in the US can be directly traced back to these early days and reminds us that even our local science culture can be shaped by the personality and priorities of distant scientists with a bully pulpit.

Weldon and Bateson were, as most scientists are, fanatical truth-seekers who shared the radical belief that nature’s truths are discoverable. Their legacy demonstrates that despite the nobility and beauty of this shared journey, we move slowly, hampered by the ignoble baggage that complicates our relationships. The lesson from this story is simple, but not easy. Science without humility and community slows our collective understanding. The history of science in the Western academy is fraught with stories like this one. In Western cultures, we often attribute discovery to individuals instead of communities, effectively making scientific discovery appear highly personal and individualistic. Furthermore, our competitive instincts to win arguments are seeded and nurtured by academic institutions that reward “being right” and punish mistakes without acknowledging that we can rarely have the former without the latter. Further, in today’s climate of rapid-fire response on social media, the community norms for engaging in scientific debate can easily erode even further. None of us is immune to the deafening arrogance of competition or the blinding lure of peer admiration, and this individualistic culture leaves science itself vulnerable as fractures develop in the community. These fractures then slow collaboration and provide a breeding ground for disinformation campaigns, the scale of which continues to grow.

My own experience is obviously limited, and yet, there may be value in sharing that experience. After 20 years in academic science, and a range of team–science interactions, it is my observation that when a scientific community is guided by a philosophy of “open-hearted curiosity,” healthy collaborations are born and important discoveries follow. What does this mean? Unfortunately, open-heartedness (and even open-mindedness) can sometimes be perceived as a weakness, somehow lacking in scientific rigor. But rigor should not be confused for ruthlessness, and open-hearted curiosity does not imply a lack of skepticism. In fact, skepticism is inherent to curiosity. Richard Feynman once said, “Science is the belief in the ignorance of experts,” and went on to explain that scientists must be trained to “both to accept and to reject the past with a kind of balance that takes considerable skill.” I argue that curiosity is the fundamental driver of this balance. The curious person does not accept assertions without evidence, they need to see, poke, and prod the data for themselves.

Curiosity alone cannot sustain healthy collaboration, but open-heartedness can protect it from toxicity. The open-hearted scientist is willing to share what they know and willing to be open and honest about what they do not know or do not yet understand. For academicians, this strikes at the heart of a vulnerability that most achievement-oriented education systems have implicitly encouraged us to hide. How can we be “experts” in our field and admit that we are ignorant? And yet, if we agree with Feynman’s astute observation of science, we are compelled to so. From an open-hearted perspective, we can shed the judgement of our ignorance, and instead appreciate that it is the raw material of future discovery. The wonderful thing about this approach is that it further compels us to question everything from our own motivations to our understanding of the data in front of us. Open-hearted curiosity provides a safe space for us to deepen our questioning. The open-hearted person further recognizes that good ideas can come from anywhere, listens without prejudice, and values the humanity of those around them. Indeed, approaching scientific problems with open-hearted curiosity is a rigorous practice that requires a great dose of humility and relentless perseverance.

Could Bateson and Weldon have benefited from open-hearted curiosity? Theirs was an era of robust debate and independent science. Their debates took on a sporting quality, as exemplified during the 1904 conference. But this model of scientific discourse may have been doomed from the beginning. After all, in the end, debate for sport is unambiguously about winning. Each side digs their heels in and aims to persuade as many people as possible. There is very little room for ambiguity and admitting any vulnerability or questioning one’s own view is a game-ending move in a sporting debate. But in a culture that explicitly values the search for truth, and not the ego of the scientist, *scientific* debate has an entirely different quality and outcome. It is no longer a sport with a single dominating champion. It becomes a scaffolded work of art, an improvisational jazz piece composed through individual creativity and communal effort. In this style of debate, parties lock intellects and together come to a deeper understanding of the world around them. In retrospect, I wonder how far Weldon and Bateson, together, could have pushed the field if they had matured in a climate of open-hearted curiosity.

When we engage each other with ego, we risk contaminating the worthiness of our shared pursuit with our own self-interests. On the other hand, if we can adopt a cultural expectation of open-hearted curiosity and learn to recognize and soothe our own insecurities, if we can allow ourselves to be vulnerable about what we do not know, if we can trust each other to share honestly and without judgement or pretense, we can create a principled scientific community deserving of discovery. We have the awesome responsibility of understanding and communicating the stuff that makes us human. These are bigger questions than we can fathom alone, and we are never so worthy to learn the secrets of the universe as when we humble ourselves to their complexity.
